# Outcome of radical prostatectomy in primary circulating prostate cell negative prostate cancer

**DOI:** 10.3332/ecancer.2016.671

**Published:** 2016-09-01

**Authors:** Nigel P Murray, Sócrates Aedo, Eduardo Reyes, Cynthia Fuentealba, Omar Jacob

**Affiliations:** 1 Hospital Carabineros of Chile, Nunoa, 7770199 Santiago, Chile; 2 Faculty of Medicine, University Finis Terrae, Providencia, 7501015 Santiago, Chile; 3 Faculty of Medicine, University Diego Portales, Manuel Rodrıguez Sur 415, 8370179 Santiago, Chile

**Keywords:** personalised medicine, genomics, biomarkers, targeted therapy, sequencing

## Abstract

**Introduction:**

Around 90% of prostate cancers detected using the serum prostate specific antigen (PSA) as a screening test are considered to be localised. However, 20–30% of men treated by radical prostatectomy experience biochemical failure within two years of treatment. The presence of primary circulating prostate cells (CPCs) in the blood of these men implies a dissemination of the tumour and could indicate a greater risk of treatment failure.

**Objective:**

To evaluate the use of the number of primary CPCs detected before surgery in the prediction of biochemical failure at ten years.

**Hypothesis:**

The dissemination of cancer cells to distant sites will determine the patient’s prognosis. The absence of primary CPCs in men undergoing radical prostatectomy for prostate cancer may imply a less aggressive disease and therefore could be utilised as a prognostic factor to predict biochemical failure after surgery.

**Methods and patients:**

A single-centre observational study of a cohort of 285 men who underwent radical prostatectomy as monotherapy for prostate cancer, in whom the number of CPCs prior to treatment was determined, and who were followed up for ten years to determine biochemical failure. A Cox proportional risks with polynomial fractions analysis was used to predict biochemical failure based on the number of primary CPCs detected. A decision curve analysis was performed for the model obtained.

**Results:**

Kaplan–Meier curves for biochemical free survival at ten years was 47.34% (95% CI 38.71–55.48%). It is important to note that in CPC negative men, the ten years Kaplan–Meier biochemical-free survival was 90.35% (95% CI 75.0–96.27) whereas in men who were primary CPC positive, the biochemical free survival rate was 30.00% (95% CI 20.34–40.60%).

The Coxs´model to predict biochemical failure using transformed data with a power of minus one for the number of primary CPCs detected, showed a Harrell´s C concordance index of 0.74 and a decision analysis curve showing a net benefit of CPC detection over other risk factors to predict biochemical failure.

**Conclusions:**

The number of primary CPCs detected before surgery permits a good prediction of subsequent biochemical failure in men undergoing radical prostatectomy as monotherapy for prostate cancer. Men negative for primary CPCs have a biochemical-free survival of over 90% at ten years and should be considered for curative surgery.

## Introduction

Prostate cancer is the second commonest cause of cancer death in the Chilean male population [1]. The number of men with prostate cancer is increasing worldwide with the demographic changes and an ageing population. In the era of prostate cancer screening using serum PSA, 90% of detected cancers are considered to be localised at the time of diagnosis [2]. However, 20–30% of these patients experience biochemical failure, usually in the first two years after surgery [3].

Early in prostate cancer, there are at least one sub-population of cancer cells that disseminate firstly to the neurovascular structures and then into the circulation [4]. The majority of these cells are eliminated by host defense mechanisms or destroyed by shear forces as they circulate in the blood and lymph systems [5]. These cells, defined as primary circulating prostate cells (CPCs), are not found in small volume low-grade cancers [6], Thus absence of these circulating cells may indicate that the patients in whom there is little or no dissemination of prostate cells, removal of the primary tumour would be curative.

We present a study of the detection of primary CPCs and the results of radical prostatectomy at five and ten years with respect to patients negative and positive for primary CPCs.

## Patients and methods

An observational study on 285 men who underwent radical prostatectomy as monotherapy for prostate cancer performed in the Hospital de Carabineros de Chile between 2005 and 2015. The study was approved by the Local Ethics Committee and complied with the Declaration of Helsinki.

After written informed consent, an 8 mL venous blood sample was taken for CPC detection (see below for details).

For each patient the following were registered; A) from the biopsy: Gleason score, number of core samples positive for cancer, and percent of core sample infiltrated, prostate volume as determined by TRUS at the time of biopsy. B) from the surgical specimen: Gleason score; presence of extracapsular extension (ECE); presence of positive surgical margins (SM) defined as one with cancer cells in contact with the inked surface of the specimen; infiltration of the seminal vesicles (SVI), and infiltration of the lymph nodes (LNI). The pathological study of the biopsy and surgical specimenswere performed by dedicated genitourinary pathologists. C) Date of prostatectomy; age and total serum PSA (ng/mL) at the time of diagnosis using the Siemens Advia Centaur XR® assay. D) pathological stage according to TNM classification

## Definition of Epstein criteria for active observation

Patients who had a biopsy confirmed prostate cancer, with a Gleason score of ≤ 6, ≤ 2 cores positive for prostate cancer, ≤ 50% infiltration in any one core, and PSA density < 0.15ng/mL/mL fulfilled the Epstein criterion for active observation [7].

## Detection of primary circulating prostate cells

Before the surgery all men had a 8 mL venous blood sample taken and collected in a tube containing ethylenediaminetetraacetic acid, EDTA, (Becton Dickinson-Vacutainer®). Samples were maintained at 4º C and processed within 48 hours. CPC detection was independently evaluated with the evaluators being blinded to the clinical details.

## Collection of CPCs

Mononuclear cells were obtained by differential centrifugation using Histopaque 1077 (Sigma-Aldrich), washed, and re-suspended in a 100 μL aliquot of autologous plasma. A 25 μL aliquots were used to make slides (silanised, DAKO, USA), were dried in air for 24 hours and fixed in a solution of 70% ethanol, 5% formaldehyde, and 25% phosphate buffered saline (PBS) pH 7.4 for five minutes and finally washed three times in PBS pH 7.4.

## Immunocytochemistry

**Figure 1.**
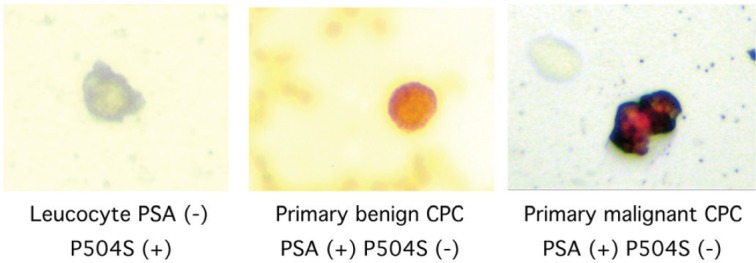
Photomicrographs of CPCs.

CPCs were detected using a monoclonal antibody directed against PSA, clone 28A4 (Novocastro Laboratory, UK), and identified using an alkaline phosphatase-anti alkaline phosphatase based system (LSAB2, DAKO, USA), with new fuchsin as the chromogen. Positive samples underwent a second process with anti-P504Sclone 13H4 (DAKO, USA) and were identified with a peroxidase based system(LSAB2,DAKO, USA) with DAB (3,3diaminobenzidinetetrahydrochloride) as the chromogen. A primary CPC was defined according to the criteria of ISHAGE (International Society of Haemotherapy and Genetic Engineering) [8], and the expression of P504S defined according to the Consensus of the American Association of Pathologists [9]; as a cell expressing both PSA and P504S and detected before definitive treatment for prostate cancer. A test was considered positive for primary CPCs when at least 1 cell/8 mL of blood was detected; the number of CPCs detected/8 mL blood sample was registered (Figure 1).

## Follow-up

All men were followed up with serial total serum PSA measurements; three monthly for two years, then six monthly to detect the presence or absence of biochemical failure (BF) for up to ten years. BF was defined as a serum total PSA > 0.2ng/mL on two separate occasions, taken at least two weeks apart.

## Statistical analysis

The program Stata (Stata/SE 14.0 for Windows, Stata Corp Lp, 2015) was used to analyse the data, describing according to the nature and distribution of the variables quantitative and ordinal for measures of central tendency (mean and median) and their distribution using inter-quartile range (IQR) and standard deviation (SD). The null hypothesis of a normal distribution of the variables was assessed using the Shapiro-Wilk test [10]. The nominal dichotomous variables were described as proportions with their respective confidence intervals [10].

The correlation of Spearman and a scatter diagram were used to analyse the number of CPCs detected. It took into consideration the variables, age, total serum PSA, and the number of biopsy core samples positive for cancer. In addition, a bi-serial correlation were performed. This was between the number of CPCs detected and the dichotomous variables such as Gleason score > 7, presence of extracapsular extension, and the pathological stage T1 or T2.

The ten-year follow-up biochemical free failure survival was described using the non-parametric model of Kaplan–Meier [11]. To construct the Cox´s model, the multivariable fractional polynomial assessment was used to establish a functional form for the nCPC, represented by the formula, [(nCPC + 1) / 10]^-1^ . The transformed value of nCPC allows a better adjustment and specification for the Cox proportional hazards regression model. Thus the regression models incorporating nCPC use the functional form (transformed variable) [11].

The model was tested for compliance with the Cox proportional hazards model using log–log plots, the Therneau and Grambsch test, and testing for a cohort time interaction. The correct specification for the respective model was done using the Linktest (10–12). In addition, for each model the log likelihood (LL), Akaike Information Criterion (AIC), Bayesian Information Criterion (BIC), and Harrell´s C test were used to predict biochemical free failure [11]. Finally with respect to the presence or absence of CPCs, the predictive model was compared with the data observed in the Kaplan–Meier curves with regards to the biochemical failure free survival at ten years [11].

## Results

Table 1 shows the clinical and pathological results of the 285 men treated with radical prostatectomy. It was seen that for all the variables, the Shapiro-Wilk test had a p-value of < 0.15.

The presence of at least one CPC/sample was seen in 224 patients (78.60%; 95% CI 73.83–83.36%). The presence of CPCs was associated with the total serum PSA and the number of positive biopsy cores (Spearman co-efficient 0.33, 0.34 respectively p < 0.01) but not with age (Spearman co-efficient -0.03 p > 0.05). The correlation bi-serial between the number of CPs detected was significantly associated with a Gleason score > 7, the presence of extra-capsular extension, and pathological stage pT1 and pT2 (Spearman co-efficient of 0.38, 0.32, and –0.33 respectively p < 0.01)

**Figure 2.**
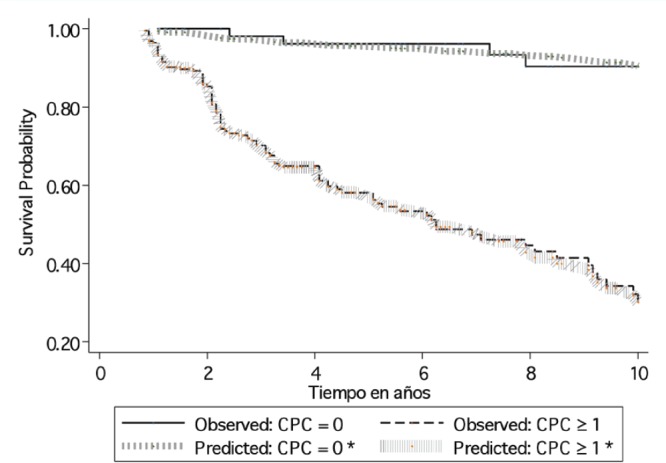
Ten year biochemical failure free survival formen positive and negative for primary CPCs, comparing observed survival (Kaplan–Meier) with respect to predicted survival (Cox model based on the number of CPCs detected*) in 285 men treated with radical prostatectomy. CPCs = primary circulating prostate cells; *based on the Cox model of proportional risks, incorporating a functional form of the variable number of CPCs (nCPC).

For the whole study population the BF free survival at ten years based on the Kaplan–Meier was 47.34% (95% CI 38.71–55.48%). In the group of patients primary CPC negative, the BF-free survival at ten years using Kaplan–Meier was 90.35% (95% CI 75.80–96.37%) compared with those men with at least one CPC detected which showed Kaplan–Meier estimation at ten years of 30.00% (95% CI 20.34–40.60%). Figure 2 shows the Cox regression model predicted survival curves versus the observed Kaplan–Meier curves for men with and without detectable CPCs. There was concordance of the two predictive models, the Cox model showing a hazard ratio of 0.70 (95% CI 0.62–0.78 p < 0.01) and a Harrell`s C of 0.74, which is classified as good in terms of prediction. Table 2 shows the Kaplan–Meier and Cox results for survival at three, five, and ten years according to the number of CPCs detected/sample.

With an increasing number of CPCs/sample detected there was a decreased BF-free survival.

## Discussion

To identify patients in whom primary treatment could be curative, a series of nomograms [14] or risk classifications [15] using pre-treatment clinicopathological findings has been proposed to stratify patients into prognostic groups. Pre-treatment findings include total serum PSA, biopsy Gleason score, number of biopsy cores positive for cancer, and percentage of core infiltration. However, more than one third of patients were undergraded based on the prostate biopsy results [16]; the Prostate Cancer Research International Active Surveillance (PRIAS) study [17] reported upstaging to pT3 in 21% of cases, a Gleason score of > 6 in 45% of cases, and with approximately 20% of these men having an unfavourable outcome after radical prostatectomy [18].

However, the success or failure of local curative treatment will ultimately depend on whether or not cancer cells have disseminated, implanted, and survived, establishing thereby that a micro-focus of tumour cells are outside the reach of local therapy. The concept of primary CPCs, those disseminating prior to primary therapy has been reported to be useful as a sequential test to detect prostate cancer [19]. It is recognised that not all primary CPCs will survive; the majority being eliminated by host defense mechanisms. In patient’s who are primary CPC negative, the inference is that because of tumour cell characteristics there is a failure in dissemination or of distant implantation, thus the tumour is truly localised to the prostate and therefore complete tumour removal would ‘cure’ the patient. It must be recognised that whatever method of detecting CPCs impose limitation on detection, and/or the biomarker selected to detect CPCs may not be capable of detecting all tumour cells, as has been reported with the use of Epithelial Cell Anchor Molecule (EpCAM) [20].

The use of primary CPC detection to stratify patients identified a group with >90% BF-free survival at ten years. These primary CPC negative patients had a significantly higher cure rate after radical prostatectomy than their primary CPC positive counterparts. The higher the number of CPCs detected/sample the worse is the survival rate, however, it must be emphasised that the test was designed to give a positive/negative result only. This is because being a manual method the inter-observer variation is relatively high, and thus it was considered to be inappropriate to define a test by the absolute number of cells detected. When considered as a positive/negative test this variation was minimal. This was seen when we used a continuous variable and compared it with the CAPRA score to predict outcome after radical prostatectomy: there was little improvement in the prognostic ability of CPCs [21]. This was different for secondary CPCs detected after radical prostatectomy. This secondary CPCs represent minimal residual disease and are associated with predicting BF-free survival [22].

The decision to treat and not to observe the patient is a tradeoff between achieving a possible cure versus the effects on the quality of life (QoL) of the treatment. PSA screening has led to early stage diagnosis and fewer patients with stage IV disease. It is noted that a total 5.8 additional cases of early stage disease are being diagnosed and 3.9 cases are being treated for each fewer case of stage IV disease [23]. The use of the presence or absence of primary CPCs to establishment prognosis could improve patient selection and guide treatment decisions.

## Conclusion

The presence or absence of primary CPCs detected before surgery permits a good prediction of subsequent BF in men undergoing radical prostatectomy as monotherapy for prostate cancer. Men negative for primary CPCs have a BF-survival of over 90% at ten years and should be considered for curative surgery.

## Conflict of interests

The authors report no conflicts of interest.

## Funding

The study was supported by a Hospital de Carabineros Research Grant.

## Figures and Tables

**Table 1.** Clinical and histological characteristics in 285 men treated with radical prostatectomy.

Variable	Descriptive statistic
Age (years) Mean ± Ds	65.86 ± 8.81
PSA (ng/mL) Median; IQR	5.84; 3.88
Number of CPCs Median; IQR	3;7
Score Gleason > 7 points % (CI95%)	35.09 (29.55–40.94)
Number of biopsy cores Median; IQR	20;30
Presence Extra-capsular extension % (CI95%)	46.67 (40.76–52.64)
Clinical stage T1 or T2 % (CI95%)	77.54 (76.70–82.39)

PSA = prostate specific antigen at the time of diagnosis; SD = standard deviation; IQR = interquartile range; CPC = primary circulating prostate cells; age showed a symmetrical distribution; The number of CPCs and biopsy cores, as well same PSA showed a skewed distribution

**Table 2.** Three, five, and ten years BF-free survival for men according to the number primary circulating prostate cells (CPCs), comparing observed survival (Kaplan–Meier) with respect to predicted survival (based on the Cox model of proportional risks, incorporating a functional form of the variable number of CPCs ) in 285 men treated with radical prostatectomy.

Number of CPCs	n	Observed Survival Kaplan–Meier			Predicted survival Cox model		
					Three year	Five year	Ten year
		Three year %;CI:95%	Five year %;CI:95%	Ten year %;CI:95%			
0	61	98.11; 87.35-99.73	96.11; 85.32–99.01	90.35; 75.80–96.35	98.10	97.09	92.90
1	16	93.75; 63.23–99.10	82.03; 42.31–95.54	82.03;42.31–95.54	88.88	83.43	63.61
2	59	85.97; 72.65–93.10	77.94; 62.43–87.65	42.95;23.98–60.64	80.59	71.77	43.68
3	8	87.5038.70–98.14	58.33; 7.65–89.31	58.337.65–89.31	74.67	63.84	32.61
4	33	80.81; 62.09–90.91	75.04;53.35–87.70	0	70.46	58.39	26.09
5	1	100	100	0	67.36	54.48	21.95
6	7	62.50; 14.19–89.31	0	0	65.00	51.58	19.14
7	1	0	0	0	63.15	49.34	17.13
8	41	58.30; 40.90–72.21	48.59; 29.87–64.97	30.37; 10.37–53.43	61.66	47.57	15.64
10	1	100	100	0	59.44	44.96	13.58
12	2	50.00;0.6–91.04	50.00; 0.6–91.04	0	57.85	43.12	12.24
15	1	0	0	0	56.17	41.22	10.94
16	24	69.18; 45.79–84.04	50.59; 26.37–70.58	0	55,74	40.73	10.62
24	10	70.00; 32.87–89.19	36.00; 8.99–64.84	0	53.48	38.22	9.06
25	1	0	0	0	53.30	38.02	8.94
28	1	100	100	0	52.81	37.49	8.63
30	1	0	0	0	52.54	37.19	8.46
32	10	40;12.27–67.02	40; 12.27–67.02	0	52.30	36.93	8.31
40	6	16.67; 7.7–51.68	16.67; 7.7–51.68	16.67; 7.7–51.68	51.57	36.14	7.88
64	1	0	0	0	50.45	34.94	7.24

CI = confidence interval; CI of survival function is not possible, calculated for value zero and 100%
